# Paternal but not maternal age influences early-life performance of offspring in a long-lived seabird

**DOI:** 10.1098/rspb.2015.2318

**Published:** 2016-04-13

**Authors:** Rémi Fay, Christophe Barbraud, Karine Delord, Henri Weimerskirch

**Affiliations:** Centre d'Etudes Biologiques de Chizé, UMR 7372 CNRS/Univ La Rochelle, Villiers-en-Bois 79360, France

**Keywords:** ageing, capture–mark–recapture, *Diomedea exulans*, juvenile survival, long-term effects

## Abstract

Variability in demographic traits between individuals within populations has profound implications for both evolutionary processes and population dynamics. Parental effects as a source of non-genetic inheritance are important processes to consider to understand the causes of individual variation. In iteroparous species, parental age is known to influence strongly reproductive success and offspring quality, but consequences on an offspring fitness component after independence are much less studied. Based on 37 years longitudinal monitoring of a long-lived seabird, the wandering albatross, we investigate delayed effects of parental age on offspring fitness components. We provide evidence that parental age influences offspring performance beyond the age of independence. By distinguishing maternal and paternal age effects, we demonstrate that paternal age, but not maternal age, impacts negatively post-fledging offspring performance.

## 1. Introduction

Heterogeneity in demographic traits within populations has strong implications for both long-term evolutionary processes and actual population dynamics [1]. Today, there is increasing evidence that conditions experienced during development explain an important part of the heterogeneity in demographic traits observed after independence [2,3]. Interactions between parental genotype, phenotype, and environment may strongly influence offspring realized fitness independently of its own genome. This source of non-genetic inheritance, called parental effects [4], is a consequence of a large variety of mechanisms ranging from DNA methylations to parental behaviours such as parental favouritism, with profound impacts for future offspring performance. Such parental effects, which are at the interface of developmental, ecological, and evolutionary processes, are fundamental mechanisms to understand individual variability in demographic traits [5].

In iteroparous species, parental age strongly influences reproductive success [6]. Variation in breeding success resulting from individual changes typically follows a dome-shaped curve pattern along the parental life [7], due to accumulating experience in early life [8] and senescence at older age [9]. Studies have shown that the variation in parental breeding abilities have important consequences for offspring quality, especially for older parents in relation to reproductive senescence [10,11]. Although there is evidence that parental age impacts negatively offspring lifespan, referred to as the Lansing effect [12], the relative effects of parental age on different offspring fitness components remain little documented in wild populations (table 1). Yet, accurate estimation of demographic traits is important to disentangle different processes implying different biological mechanisms which can lead to the same general pattern [16]. For example, the reduced life span of individuals from old parents may emerge from decreasing survival probability through the lifetime or accelerating senescence rate in late life. However, few studies are able to assess accurately offspring performance after independence. Furthermore, studies on parental effects are biased toward maternal effects (table 1), although it was recently recognized that both maternal and paternal effects are common and important in a large variety of organisms [22]. In the wild, assessing the effects of parental age on offspring performance requires high-quality longitudinal datasets with known age individuals and pedigree information. Such conditions are rare due to technical and financial limitations, which explain why evidence of delayed parental effects on offspring performance in wild populations remains limited to date.

**Table 1.** Review of studies investigating the effect of parental age on offspring performance after independence in wild populations.

species	offspring trait	paternal age effect	maternal age effect	reference
birds				
blue footed booby *Sula nebouxii*	recruitment rate	qua	qua	Torres *et al*. [13]
great tit *Parus major*	age at last reproduction	/	no (f)	Bouwhuis *et al*. [14]
great tit *Parus major*	LRS	/	no (f)	Bouwhuis *et al*. [14]
red-billed chough *Pyrrhocorax pyrrhocorax*	juvenile survival^a^	no	no	Reid *et al*. [15]
common tern *Sterna hirundo*	LRS	− (m) no (f)		Bouwhuis *et al*. [16]
common tern *Sterna hirundo*	lifespan	− (m) no (f)	no (m, f)	Bouwhuis *et al*. [16]
house sparrows *Passer domesticus*	LRS	− (m) no (f)	− (f) no (m)	Schroeder *et al*. [17]
house sparrows *Passer domesticus*	lifespan	no (m, f)	no (m, f)	Schroeder *et al*. [17]
mammals				
red squirrel *Tamiasciurus hudsonicus*	juvenile survival^a^	/	qua	Descamps *et al*. [11]
Weddell seals *Leptonychotes weddellii*	survival to maturity^a^	/	+ (f)	Hadley *et al*. [18]
Weddell seals *Leptonychotes weddellii*	recruitment probability	/	− (f)	Hadley *et al*. [18]
European rabbit *Oryctolagus cuniculus*	survival to maturity	/	+ (f)	Rodël *et al*. [19]
European rabbit *Oryctolagus cuniculus*	LRS	/	qua (f)	Rodël *et al*. [19]
rhesus macaque *Macaca mulatta*	juvenile survival	/	− (m, f)	Hoffman *et al*. [20]
rhesus macaque *Macaca mulatta*	age at first reproduction	/	no (f)	Hoffman *et al*. [20]
rhesus macaque *Macaca mulatta*	reproductive rate	/	no (f)	Hoffman *et al*. [20]
preindustrial humans *Homo sapiens*	LRS	/	− (m, f)	Gillespie *et al*. [21]
preindustrial humans *Homo sapiens*	survival to maturity	/	qua (m, f)	Gillespie *et al*. [21]
preindustrial humans *Homo sapiens*	recruitment probability	/	− (m, f)	Gillespie *et al*. [21]

^a^Apparent survival. For recruitment trait, recruitment rate, confounding early-life survival with recruitment, is distinguished from recruitment probability which is conditional on survival until maturity. LRS indicates lifetime reproductive success. Paternal and maternal age can have no effect (no), linear negative effect (−), linear positive effect (+), positive quadratic effect (qua), or is not assessed (/). For studies distinguishing male (m) from female (f) offspring performance, sex-specific effects are indicated.

In this study, we investigated the effects of both paternal and maternal age on the long-term post-fledging offspring performance of the wandering albatross *Diomedea exulans*. In this species, age affects parental care with strong senescence effects [23–25]. Delayed effect of parental age on offspring performance was assessed through juvenile survival, recruitment probability, and age of recruitment. First, due to senescence in male and female breeding performance, we predicted that parental age should be negatively related to offspring survival and recruitment. Second, knowing that parental care during chick rearing is strongly biased toward males [26], and that senescence effects are stronger for males than for females in wandering albatross [24], we predicted a larger effect of paternal age on offspring performance than of maternal age.

## 2. Material and methods

### (a) Study species and field method

The study was conducted on Possession Island (46.8° S, 51.8° E), Crozet Island in the Indian Ocean, where long-term monitoring of wandering albatrosses, based on annual capture–mark–recapture methodology, has been carried out annually since 1960. Sex assignments were performed based on both field observations (i.e. sexual size and plumage dimorphism, mating behaviours) and genetic analyses since 1999 (electronic supplementary material, appendix S1). Wandering albatrosses show a typical slow life-history strategy with high adult survival rates and low productivity (i.e. quasi-biennial reproduction and clutch size limited to one egg without replacement laying). The period of parental care is especially long, lasting about 10 months, from laying in January to fledging in November. Parental care is shared by both sexes although male involvement is more important during chick rearing [26]. Fledglings do not receive post-fledging care and leave the colony alone remaining at sea continuously for the following 2–7 years.

### (b) General model

Individual encounter histories were modelled using a multi-event approach. The model consisted of seven states, one immature state, five adult states and the state dead (electronic supplementary material, figure S1), and five events. To consider individuals during the period of immaturity, we defined the Pre-Recruitment state (PrR) after which immature birds can recruit, i.e. lay an egg for the first time into the breeding population. Adult birds can transit toward Successful Breeder state (SB), when the chick fledged, Failed Breeder state (FB), when the chick died before fledging, or recruited Non-breeder state (NB), when individuals that have recruited in the population (i.e. bred at least once) were observed as non-breeders at the colony. To model the sabbatical years spent continuously at sea after reproduction, we added two unobservable states [27] corresponding to the two previous breeding states defined: Post-successful Breeder (PSB) and Post-failed Breeder (PFB). Thus, adults that are at sea (i.e. not at colonies for a whole year) are distinguished based on their most recent breeding state last time they were at a colony. In our study, state assignment was not always certain since between 1966 and 1986, as state assessment was unknown for a number of breeders; some individuals were classified as breeders but the success or failure was not always ascertained. Multi-event models allowed us to deal with state uncertainty by assessing the likelihood of an individual state given the events (i.e. observations) [28]. We considered five events, i.e. five types of observation in the field: 0, ‘not observed’; 1, ‘seen as non-breeder’; 2, ‘seen as a failed breeder’; 3, ‘seen as a successful breeder’; 4, ‘seen as a breeder but successful status not ascertained’. Our model allowed us to estimate the probability of survival (*φ*), the probability of recruitment given survival (*ψ*^rate^) and the probability of early recruitment given recruitment (*ψ*^early^). Early recruitment was defined as first reproduction occurring before eight years old for females and nine years old for males corresponding for both sexes to the first quartile of age recruitment frequency distributions [29]. Details of the parametrization with the biological constraints applied can be found in electronic supplementary material, appendix S2.

### (c) Parental age and model selection

For our analyses, we used the capture histories of 4 538 chicks from 1 107 fathers of known age (from six to 41 years old with an average of 4.1 chicks per father) and of 4 294 chicks from 1 060 mothers of known age (from six to 45 year old with an average of 4.0 chicks per mother; electronic supplementary material, figure S2). Among those chicks, 3 454 came from known aged mothers and fathers (electronic supplementary material, figure S3). All chicks were ringed and resighted between 1977 and 2013. Impact of parental effects was assessed on juvenile survival, i.e. the two first years of life from fledging, probability of recruitment, and probability of early recruitment. We fitted the logistic model: logit(*Φ*) = *β*_0_ + *β*_1_ × *x_i_*_,_ where *Φ* is a demographic parameter, *β*_0_ is an intercept parameter, *β*_1_ is a slope parameter, and *x_i_* is the age of the parent of individual *i* at birth. We tested both linear and quadratic relations owing to the dome-shaped curve pattern observed for breeding success variation with age [30]. When the parental age effect was supported, we tried, as a second step, to fit threshold models (electronic supplementary material, table S1). In our dataset, different offspring could have the same parents introducing non-independence between individuals. To check the robustness of the results, models that provided a better fit with the parental age effect (ΔAIC > 2) were run a second time with parental identity as a random effect. We calculated a likelihood ratio test (LRT) between a model where the demographic trait was constant [*φ*(cst + random(PI)] and a model where the demographic trait was a function of parental age (*a*) [*φ*(*a* + random(PI)], considering for both parental identity (PI) as a random effect. The *p*-value of the LRT corresponded to halving the *p*-value from using a *χ*² distribution with 1 d.f. [31]. This random effect was not used systematically in our model selection due to the large computation time required. We tested the effect of paternal, maternal, and average age of both parents on offspring performance. In wandering albatross, pair bonds usually last until one member of the pair dies. Thus, when individuals grow older, the age effect could be confounded with the effect of age difference within a breeding pair. To avoid spurious conclusions, we tested if the age difference within a pair provided better support to explain early life offspring performance.

Between-individual heterogeneity may lead to detecting an effect of an individual covariate more often than it should be [32]. To take into account individual variation that cannot be measured, models that provided a better fit with parental age effect (ΔAIC > 2) were run a second time with individuals as random effects on the focus demographic trait [31]. To test for the hypothesis of unaccounted individual heterogeneity on the demographic trait, we calculated a LRT between a model where the demographic trait was constant [*φ*(cst)] and a model where the demographic trait was modelled as a function of heterogeneity [*φ*(cst + *h*)], where *h* indicates between-individual heterogeneity. To test for the hypothesis of an effect of parental age (*a*) on the demographic trait while accounting for heterogeneity we calculated a LRT between a model where the demographic trait was modelled as a function of heterogeneity [*φ*(cst + *h*)], and a model where the demographic trait was modelled as a function of parental age and heterogeneity [*φ*(*a* + *h*)]. Finally, we estimated the importance of within-individual effects (i.e. individual deterioration due to senescence) and between-individual effects (i.e. progressive appearance or disappearance of individuals) in the observed pattern using the methodology described by van de Pol & Wright [33].

All models were run using the program E-SURGE [34]. There is no test available to assess the goodness of fit (GOF) of multi-event models. Hence, we performed GOF tests using the program U-CARE [35] on a simplified dataset which distinguished solely the successful breeders and failed breeders and assigning randomly a reproductive status, i.e. failed or successful, to each individual for which no information was available.

## 3. Results

**Figure 1.**
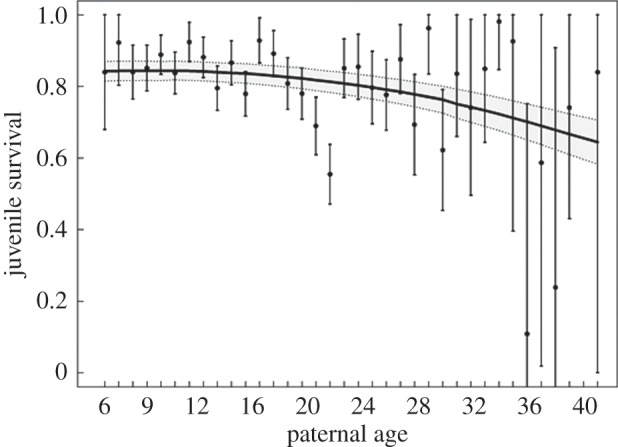
Relationship between juvenile survival and paternal age. The plain line represents the predicted relationship obtained from a model averaging procedure on constant, linear, quadratic, and all tested threshold models (table 2; electronic supplementary material, table S4). For illustration the relationship is shown for female juvenile survival. Dashed lines and bars indicate ±s.e.

GOF tests (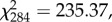 *p* = 1.00 and 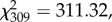 *p* = 0.75 for females and males from known paternal age, respectively, and 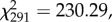 *p* = 1.00 and 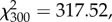 *p* = 0.51 for females and males from known maternal age, respectively) indicated that the general model fitted the data correctly (electronic supplementary material, table S2). We found that juvenile survival but not recruitment varied in response to parental age. Model selection indicated a strong linear negative effect of paternal age on offspring survival (table 2, M1 versus M2: ΔAIC = 7.8), with no clear evidence for a quadratic relationship (table 2, M2 versus M4: ΔAIC = −1.5). Knowing that no chicks from fathers older than 32 years of age have been recaptured, we tested the robustness of this result by reanalysing the data after removing these individuals. Consistently, we still found support for a negative relationship between paternal age and juvenile survival (ΔAIC = 5.6). Although threshold models did not provide a better fit (electronic supplementary material, table S1), they suggested that the negative relation between paternal age and juvenile survival was less supported before 15 years. Indeed, models with a threshold at 10, 15, or 20 years old had as much support as a strictly linear model (ΔAIC < 2), and the slopes of models with thresholds at 10 or 15 years old were not meaningfully different from 0. Model averaging, accounting for uncertainty in model selection, suggested no relationship in young paternal age and a negative effect of paternal age after 20 years on juvenile survival (figure 1). This result was robust with both the addition of parental identity as a random effect (electronic supplementary material, table S3, M3 versus M2: LRT *χ*^2^_1_ = 9.5, *p* = 0.002), and the addition of a random effect to cope with unexplained variability in juvenile survival (electronic supplementary material, table S3, M5 versus M4: LRT *χ*^2^_1_ = 4.9, *p* = 0.027). This result was not induced by increasing age difference within a breeding pair with age. Indeed, this variable was less supported than paternal age only (electronic supplementary material, table S4, M1 versus M2: ΔAIC = 3) despite the high correlation between these variables (electronic supplementary material, figure S3). There was no evidence for a relationship between maternal age and juvenile survival (table 2, M5 versus M6: ΔAIC = −1.5), neither between average age of both parents and juvenile survival (electronic supplementary material, table S5, M1 versus M2: ΔAIC = −0.7). The predominant effect of paternal age was less clear-cut when considering the analysis integrating simultaneously the age of both parents (electronic supplementary material, tables S6 and S7), but this could be explained by the lower sample size used for these specific analyses, and by the difficulty to distinguish maternal from paternal age effects as parental ages are correlated due to assortative mating. Once juvenile survival was considered, we did not find any evidence of an impact of paternal, maternal, or average age of both parents on the recruitment process. The effect of paternal but not maternal age on offspring performance was supported by the observed recruitment rate in relation to parental age. This demographic parameter was negatively related to paternal but not maternal age (paternal age: slope = −0.025 ± 0.008, *p* = 0.003; maternal age: slope = −0.006 ± 0.008, *p* = 0.529; electronic supplementary material, figure S4). This transgenerational effect came from within-individual effects of senescence and not from the progressive appearance or disappearance of particular individuals (electronic supplementary material, table S8).

**Table 2.** Testing for the effects of paternal and maternal age (*a*) on early life survival (*φ*), recruitment rate (*ψ*^rate^), and early recruitment probability (*ψ*^early^) for wandering albatrosses, Possession Island, 1977–2013. *k*, number of parameters; Dev, deviance; AIC, Akaike information criterion; cst, constant; *a*, age of the parent; *a*. sex, interaction between age of the parent and sex of the juvenile. Best supported models are in bold characters.

no.	model	*k*	Dev	AIC	slope**±**s.e.
*survival*					
	*paternal age*				
M1	*φ*(cst)	68	28 597.4	28 733.4	
**M2**	****φ******(*****a*****)**	**69**	**28 587.6**	**28 725.6**	**−0.17 ± 0.05**
M3	*φ*(*a*. sex)	70	28 586.9	28 726.9	
M4	*φ*(*a* + *a*^2^)	70	28 587.1	28 727.1	
	*maternal age*				
**M5**	****φ******(*****cst*****)**	**68**	**27 540.2**	**27 676.2**	
M6	*φ*(*a*)	69	27 539.7	27 677.7	−0.04 ± 0.06
M7	*φ*(*a*. sex)	70	27 539.1	27 679.1	
M8	*φ*(*a* + *a*^2^)	70	27 539.7	27 679.7	
*recruitment*					
	*paternal age*				
**M12**	****ψ****^**rate**^**(*****cst*****)**	**68**	**28 597.4**	**28 733.4**	
M13	*ψ*^rate^(*a*)	69	28 597.0	28 735.0	−0.01 ± 0.06
M14	*ψ*^rate^(*a.* sex)	70	28 595.9	28 735.9	
M15	*ψ*^rate^(*a* + *a*^2^)	70	28 596.4	28 736.4	
	*maternal age*				
**M16**	****ψ****^**rate**^**(*****cst*****)**	**68**	**27 540.2**	**27 676.2**	
M17	*ψ*^rate^(*a*)	69	27 539.5	27 677.5	0.05 ± 0.06
M18	*ψ*^rate^(*a.* sex)	70	27 538.8	27 678.8	
M19	*ψ*^rate^(*a* + *a*^2^)	70	27 539.4	27 681.4	
*early recruitment*					
	*paternal age*				
**M20**	****ψ****^**early**^**(*****cst*****)**	**69**	**28 595.5**	**28 733.5**	
M21	*ψ*^early^(*a*)	70	28 595.3	28 735.3	−0.02 ± 0.09
M22	*ψ*^early^(*a*. sex)	71	28 595.1	28 737.1	
M23	*ψ*^early^(*a* + *a*^2^)	71	28 594.4	28 736.4	
	*maternal age*				
**M24**	****ψ****^**early**^**(*****cst*****)**	**69**	**27 542.4**	**27 680.4**	
M25	*ψ*^early^(*a*)	70	27 542.2	27 682.2	−0.02 *±* 0.10
M26	*ψ*^early^(*a.* sex)	71	27 542.0	27 684.0	
M27	*ψ*^early^(*a* + *a*^2^)	71	27 541.8	27 683.8

## 4. Discussion

This study of a long-lived seabird provided evidence that paternal age affects offspring fitness components after independence. Juvenile survival was negatively related to paternal age, with no effect of maternal age. Given that long-distance natal dispersal is exceptional in this highly philopatric species [36], we are confident that our apparent survival estimates are close to the true survival probabilities. Few studies have estimated offspring survival after independence in relation to parental age in wild populations, and they report contrasting results including quadratic patterns (red squirrel *Tamiasciurus hudsonicus* [11]; blue footed booby *Sula nebouxii* [13]), positive relationships (Weddell seals *Leptonychotes weddellii* [18]; European rabbit *Oryctolagus cuniculus* [19]), negative relationships (rhesus macaque *Macaca mulatta* [20]), and no relationship (great tit *Parus major* [14]; red-billed chough *Pyrrhocorax pyrrhocorax* [15]). Note that these results could be sex-specific and that some studies did not control for offspring sex (table 1). Our model selection supported continuous decreasing juvenile survival with paternal age for offspring of both sexes. This linear effect is probably more a simplification from modelling rather than the expression of a biological pattern. Indeed, for young fathers, increasing age was not clearly linked to decreasing offspring survival and was even slightly positive when considering recruitment rate, a fitness component closer to real realized fitness than juvenile survival. In addition, results obtained with threshold models suggested that juvenile survival was not affected by paternal age until 15 years old. Finally, model averaging suggested that paternal age affected strongly juvenile survival mainly after 20 years (figure 1). This pattern was very similar to the pattern observed in the same species for variation in breeding success with age [30].

The negative expected relation between parental age and offspring performance, based on the general decline in parental performance due to senescence, has been documented in laboratory species [37] and human populations [21]. Previous studies on wandering albatross have shown that ageing in this species involved deterioration in foraging ability [38] with strong consequences on reproductive performance including lower breeding probability, and given they breed, lower hatching and fledging probabilities [25]. In the wandering albatross, continuously decreasing chick pre-fledging weight has been reported to occur in relation to parental age [23]. A bad start in early life could be highly detrimental to survival over the first months after independence [26], when most of the mortality between fledging and recruitment is expected to occur [39].

A pattern observed at the population scale may not result from within-individual changes and could be produced by the selective disappearance of individuals. However, in our case, the parental age effect was most probably caused by individual changes as the ageing pattern in parental care is assumed to be shaped by within-individual processes [23]. Moreover, a recent study suggested the selective disappearance of poor-quality breeders (Fay *et al*. in press [40]) and, therefore, that long-lived parents were individuals of higher quality able to allocate more into reproduction than short-lived parents. This statement was supported by our analysis decoupling within-individual from between-individual effects, suggesting that the progressive disappearance of low-quality breeders decreases the negative effect of paternal age on juvenile survival that we observed at the population scale.

Interestingly, we found that paternal but not maternal age was related to post-fledging survival. Two complementary mechanisms can explain this pattern. First, the rate of senescence is sex-specific in wandering albatrosses, the fitness cost of senescence being 2.3 times larger in males than in females [24]. Old males forage in different waters than prime-aged males, making longer trips at sea and failing to restore baseline corticosterone levels which suggest that their level of stress remains high at old age when foraging [38]. Second, parental care is biased toward males in the wandering albatross, i.e. males perform more frequent visits provisioning more food to their offspring than females [26]. This bias may be due to sexual size dimorphism, males being larger and heavier than females [41] and sex-specific foraging areas, as males forage over the continental shelf edge while females move farther off in oceanic waters bordering the shelf edge. Thus, chick characteristics at fledging probably depend more on paternal than maternal foraging abilities.

Our results also suggest that paternal effects do not persist until recruitment. Similarly, in rhesus macaque, maternal age was negatively related to early life survival but did not affect age of first reproduction [20]. In a long-lived species such as the wandering albatross, owing to the very long period of immaturity, i.e. 9–10 years, inter-individual heterogeneity could be gradually weakened by viability selection [42].

To conclude, we found that paternal but not maternal age impacted offspring performance beyond the age of independence. Such paternal effects are rarely documented in wild populations and this result was, to our knowledge, the first to suggest a predominant effect of paternal age over maternal age. We suggest that this effect could be explained by sex-specific senescence in relation to ecological constraints. As senescence seems to be widespread in animal populations [9], we may expect transgenerational effects, having implications for both population dynamics and evolutionary processes, to be common in the wild.

## Supplementary Material

ESM_Fay.docx

## Data accessibility

The data of parental ages and identities are available at http://dx.doi.org/10.5061/dryad.8g531.
