# Supplementary material for: Paternal but not maternal age influences early-life performance of offspring in a long-lived seabird
Source: Proc Biol Sci. 2016 Apr 13;283(1828):20152318. doi: 10.1098/rspb.2015.2318 (PMC4843644; doi:10.1098/rspb.2015.2318)
Supplement: ESM_Fay.docx [file rspb20152318supp1.docx]

**Supporting information**

Online appendix S1: Field methodology and sex assignment

Each year, from early to mid-December pre-breeding adults are controlled over the whole island. From mid-January (just after egg laying is resumed) to mid-February at least 3 visits are made every 10 days to identify the two members of the pairs and their breeding status. In mid-April, June and August nests are checked and the chick status noted (alive/dead). During all visits, non-breeding individuals (mainly immatures) are searched for and their identity determined (from ring number) when possible. From mid-September to mid-October all fledglings are ringed. Chicks that died on the colony between ringing and fledging are noted during the first checks of the following breeding cycle and were excluded from our data set.

Individuals were sexed in the field based on sexual size and plumage dimorphism, courting and mating behaviours. Since 1999 genetic assignments were available and were systematically used from 2003 (Weimerskirch et al. 2005). Sex was known for 2792 birds from known paternal age (1415 females and 1377 males) and for 2612 birds from known maternal age (1333 females and 1279 males). Of the remaining unsexed birds, 96 % were never seen after fledging on Possession Island, and can be considered as dead before recruitment (Charmantier et al. 2011). Knowing that our study population showed a balanced sex ratio at fledging (*n* = 3126, *p* = 0.99) and that the sex ratio of recaptured birds was also balanced (*n* = 3085, *p* = 0.43), we deduced that the large majority of these birds never seen at the colony were individuals of both sexes in equal proportion. As all individuals needed to be included in the model in order to avoid overestimating survival, we inferred the sex of these unsexed birds using a binomial random distribution as in Pardo et al. (2013).

References

Charmantier A., Buoro M., Gimenez O. and Weimerskirch H. 2011 Heritability of short‐scale natal dispersal in a large‐scale foraging bird, the wandering albatross. *Journal of evolutionary biology* 24, 1487-1496.

Pardo D., Barbraud C. and Weimerskirch H. 2013 Females better face senescence in the wandering albatross. *Oecologia* 173, 1283-1294.

Weimerskirch H., Lallemand J. and Martin J. 2005 Population sex ratio variation in a monogamous long-lived bird, the wandering albatross. *Journal of Animal Ecology* 74, 285-291.

Online appendix S2: Parametrization of the general model and biological constraints

Several biological constraints were applied to improve parameter identifiability. The initial state was constrained to the pre-recruitment state because all birds were banded as chick. Then, from the first year of life to the fifth, transition probability to the pre-recruitment stage was fixed to 1 because no recruitment occurred before 6 year-old. To model the pre-recruitment period we defined two main stages: the *juvenile* stage, which was an unobservable state, corresponding to the first 2 years of life spent continuously at sea (i.e. no individual of 1 or 2 year-old were observed at the colony), and the *immature* stage corresponding to non-recruited birds older than 2 years that started to visit the colony and could be potentially observed. Based on previous results provided by Fay et al. (2015), the immature stage was decomposed in three age classes: 3-8 years, 8-13 years and >13 years. Juvenile survival was set to be cohort dependent and both juvenile and immature survival rates were assumed to be sex-dependent. Recapture probability of the immature stage was modelled as age dependent to fit the progressive return of individuals at the breeding colony before recruitment. From 6 years of age birds may recruit and move toward the mature states SB and FB depending both on sex and age. Recruitment rate was constrained to be constant after age 10 (Fay et al. 2015). For mature birds, survival was assumed to be sex-dependent (Barbraud and Weimerskirch 2012). Based on Pardo, Barbraud & Weimerskirch (2014), we distinguished different survival probabilities for breeders and post reproductive breeders on one side and recruited non-breeders on the other side. Note that we were not able to model actuarial senescence as in Prado et al. (2013) due to a very small sample size of old individuals with known aged parents. Transitions were set to be state dependent. Since wandering albatrosses are monogamous and both sexes exhibit a quasi-biennial breeding, transitions were constrained to be similar between sexes. Recapture probabilities were assumed to be state dependent due to lower detection probability for observable non-breeders and failed breeders compared to successful breeders. Finally, our general model was:

$${\Phi_{a_{(1to2)}.sex.cohort, a_{(3to8,9to13,13+)}.sex}^{pre}\Phi_{sex.state}^{ad}}\Psi_{a.sex}^{pre\to ad}\Psi_{cst}^{ad\to ad} p_{a}^{pre}p_{state}^{ad}$$

where the pre-recruitment (pre) survival probability (Φ) was age-class (a), sex and cohort dependent, the adult (ad) survival probability was sex and state dependent, the probability of transition (Ψ) from pre-recruitment to adult was age and sex dependent, the probability of remaining in the adult stage was constant (cst), the pre-recruitment capture probability (p) was age dependent, and the adult capture probability was state dependent. In this model notation, symbols “.” indicate interactive effects, “1to2”, “3to8” and “9to13” indicate that age classes were grouped and “>13” indicates that age classes were grouped after 13 years.

References

Barbraud C. and Weimerskirch, H. 2012 Estimating survival and reproduction in a quasi-biennially breeding seabird with uncertain and unobservable states. *Journal of Ornithology.* 152, 605–615.

Fay R., Weimerskirch H., Delord K. and Barbraud, C. 2015 Population density and climate shape early-life survival and recruitment in a long-lived pelagic seabird. *Journal of Animal Ecology* 84, 1423–1433.

Pardo, D., Barbraud, C. and Weimerskirch, H. 2013 Females better face senescence in the wandering albatross. *Oecologia* 173, 1283–1294.

Pardo D., Barbraud C. and Weimerskirch, H. 2014 What shall I do now? State-dependent variations of life-history traits with aging in Wandering Albatrosses. *Ecology and Evolution* 4, 474–487.

Online Figure S1. Life cycle graph representing transitions between observable (white) or unobservable states (grey). All birds are ringed as chicks, thus individuals start in the Pre-Recruitment state (PrR). After fledging, all birds remain at least two years continuously at sea. Pre-Recruitment state becomes observable from 3 years-old when birds start to return at the colony. From 6 years-old, birds can pass into the breeding group of the population though recruitment represented by dashed arrows. Then mature birds irrespectively of age can transit between mature states: successful breeder (SB), failed breeder (FB), recruited non breeder (NB), post successful breeder (PSB) and post failed breeder (PFB).

PrR chick

NB

SB

FB

Pre-recruitment state

PrR 1-2years

PrR 3-5years

PrR >5years

PFB

PSB

Mature states

Online Figure S2. Number of chicks from fathers (a) and mothers (b) of known age. Numbers indicate sample size when it was below 20 individuals for a given parental age.


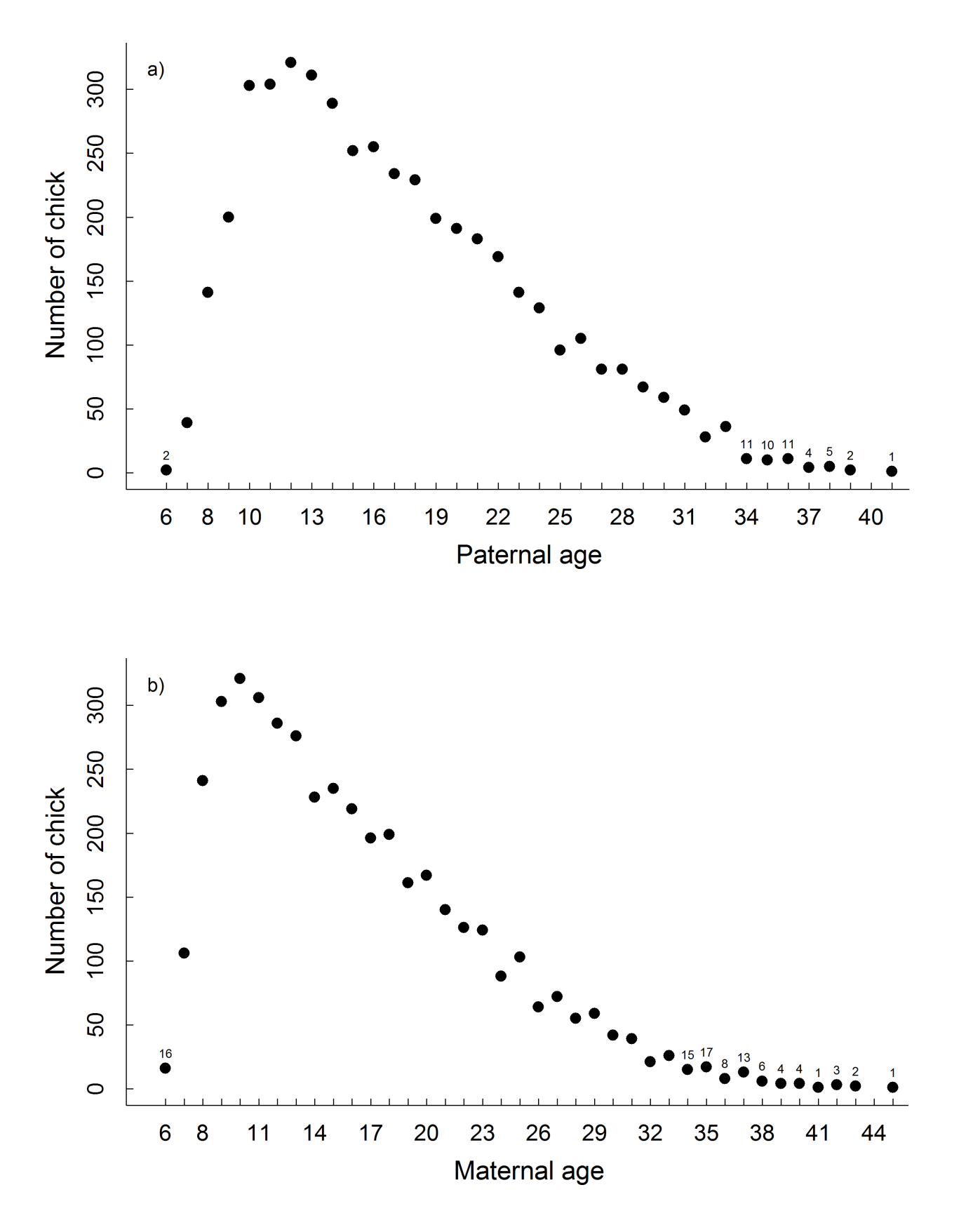


Online Figure S3. Age difference between parents in relation to paternal age. Numbers indicate sample sizes.


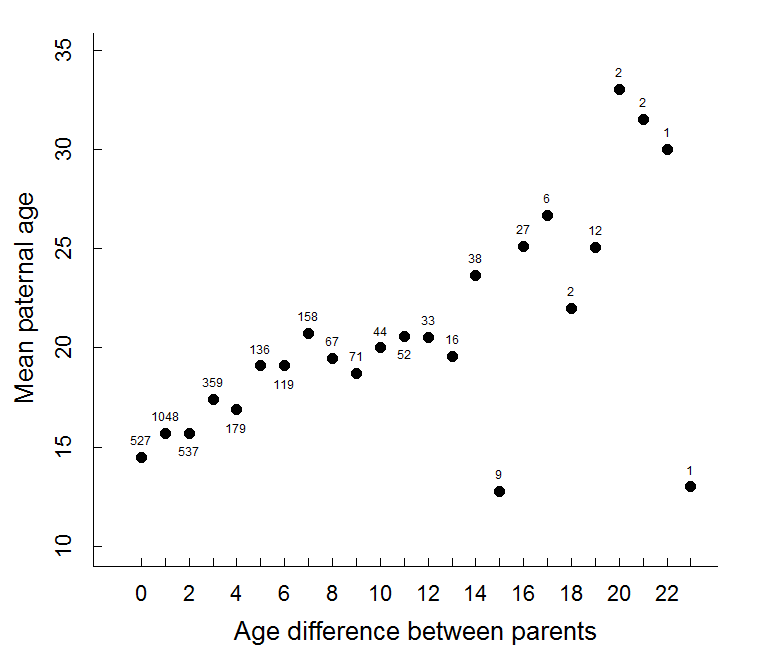


Online Figure S4. Observed recruitment rate in relation to paternal (a) and maternal (b) age. To model recruitment rate as a function of parental age, we used generalized linear mixed models (GLMM) with a binomial error-structure and a logit link function fitted to a binary response variable (recruited = 1, not recruited = 0), with parental identity as random effect. Recruit was assessed 15 years after fledging, when most of the recruitments have occurred. Plain lines represent curves derived from a model averaging procedure performed on models with constant, linear and quadratic relationships between parental age and offspring recruitment rate.


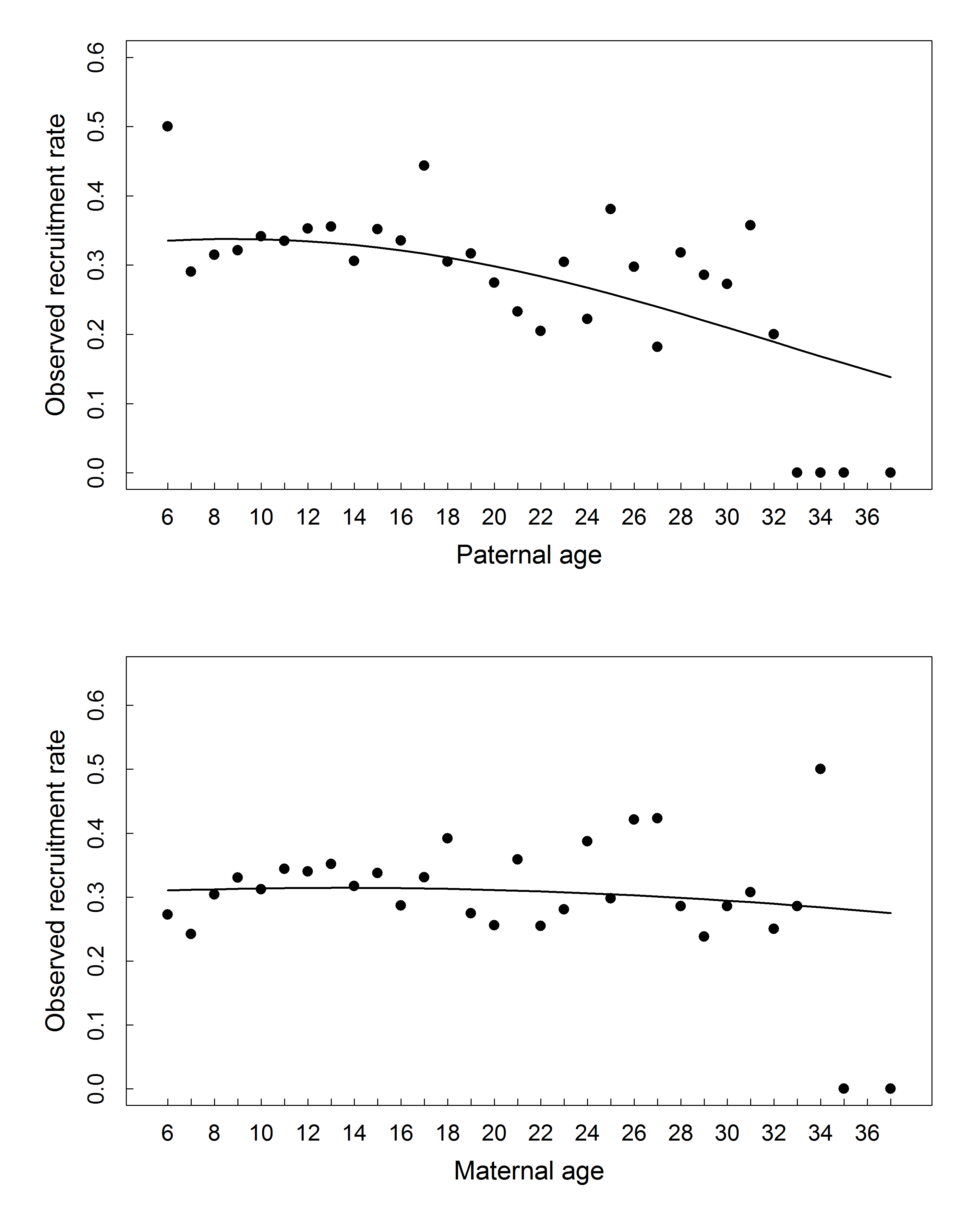


Online Table S1: Threshold model selection for the effect of paternal age (a) on early life survival (φ), for wandering albatrosses at Possession Island, 1983-2013 (n = 4538 chicks). k = number of parameters, Dev = deviance, AIC = Akaike Information Criterion. a ≤ n = function of paternal age with a threshold at n year. Threshold models were implemented fitting the logistic model: logit(Φ) = β_0_ + β_1_ * a_i≤n_ + β_2_ * a_i>n_ , where Φ is a demographic parameter, β_0_ is an intercept parameter, β_1_ and β_2_ are the slope parameters, and a_i_ is the age of the parent at birth of individual i. Thus, we fitted two linear models with one intercept. β_1_ is the slope describing the relation for juvenile survival and paternal age between 6 to n years old and β_2_ for juvenile survival and paternal age between n+1 to 41 year-old. The best supported model is in bold characters.

| No. | Model | k | DEV | AIC | Slope1 **±** s.e. | Slope2 **±** s.e. |
| --- | --- | --- | --- | --- | --- | --- |
| *Survival* |  |  |  |  |  |  |
| **M1** | $\boldsymbol{\varphi(a)}$ | **69** | **28587.6** | **28725.6** | **-0.17±0.05** | **/** |
| M2 | $\varphi(a \leq10)$ | 70 | 28586.8 | 28726.8 | -0.09 ± 0.10 | -0.20 **±** 0.06 |
| M3 | $\varphi(a \leq15)$ | 70 | 28586.7 | 28726.8 | -0.07 **±** 0.11 | -0.24 **±** 0.09 |
| M4 | $\varphi(a \leq20)$ | 70 | 28587.2 | 28727.2 | -0.11 ± 0.10 | -0.21 **±** 0.09 |
| M5 | $\varphi(a \leq25)$ | 70 | 28587.6 | 28727.6 | -0.16 ± 0.07 | -0.18 **±** 0.09 |
| M6 | $\varphi(a \leq30)$ | 70 | 28587.6 | 28727.6 | -0.15 ± 0.06 | -0.24 ± 0.13 |

Online Table S2. Detail results of GOF tests.

|  | Chicks from known paternal age | | | | | |
| --- | --- | --- | --- | --- | --- | --- |
|  | Females | | | Males | | |
| Test | χ2 | df | P | χ2 | df | P |
| WBWA | 39.933 | 52 | 0.889 | 70.993 | 63 | 0.229 |
| 3G.SR | 42.221 | 55 | 0.897 | 53.498 | 51 | 0.379 |
| 3G.Sm | 134.582 | 180 | 0.995 | 143.865 | 197 | 0.998 |
| M.ITEC | 65.410 | 31 | <0.001 | 102.275 | 39 | <0.001 |
| M.LTEC | 18.636 | 16 | 0.288 | 42.961 | 18 | 0.001 |
|  | Chicks from known maternal age | | | | | |
|  | Females | | | Males | | |
| Test | χ2 | df | P | χ2 | df | P |
| WBWA | 41.849 | 53 | 0.865 | 75.177 | 63 | 0.14 |
| 3G.SR | 31.912 | 52 | 0.987 | 64.463 | 50 | 0.082 |
| 3G.Sm | 135.355 | 187 | 0.998 | 153.241 | 190 | 0.977 |
| M.ITEC | 73.933 | 32 | <0.001 | 91.791 | 42 | <0.001 |
| M.LTEC | 21.172 | 17 | 0.219 | 24.639 | 16 | 0.076 |

GOF tests indicated that one assumption of the JMV model was not supported. Indeed, the test M.ITEC indicated the presence of trap-dependence. This effect was due to the quasi-biennial breeding strategy of wandering albatrosses. Individuals which are breeders a given year have a greater chance to take a sabbatical year spent continuously at sea the next year and such to be missed at colony. Unlike breeders, individuals which are on sabbatical year a given year have a greater chance to attempt to breed the next year and then to be observed at the colony. This behavior creates trap-shyness in GOF tests. However, given that our general model structure included unobservable states to explicitly take into account quasi-biennial breeding, we ignored the M.ITEC component in the GOF test of the general model.

Online Table S3: Testing for the effects of paternal age (a) on early life survival (φ) considering individual random effect (h) and paternal identity as a random effect (PI) for wandering albatrosses from Possession Island, 1977-2013 (n = 4538 chicks). Results include the number of parameters (k), the deviance (Dev), the Likelihood Ratio Test (LRT), and the p value (p). Best supported models are in bold characters.

| No. | Model | k | Dev | *LRT* | *p* |
| --- | --- | --- | --- | --- | --- |
|  | Parental identity random effect | |  |  |  |
| M1 | $\varphi(cst)$ | 68 | 28597.4 |  |  |
| M2 | $\varphi(cst+PI)$ | 69 | 28597.1 | 0.3 | 0.584 |
| M3 | $\varphi(a+PI)$ | 70 | 28587.6 | 9.5 | 0.002 |
|  | Individual random effect |  |  |  |  |
| M1 | $\varphi(cst)$ | 68 | 28597.4 |  |  |
| M4 | $\varphi(cst+h)$ | 69 | 28589.0 | 8.4 | 0.004 |
| M5 | $\varphi(a+h)$ | 70 | 28584.1 | 4.9 | 0.027 |

Online Table S4: Testing for the effects of age difference between the parents (dif) on early life survival (φ), recruitment rate (ψ^rate^) and early recruitment probability (ψ^early^) for wandering albatrosses, Possession Island, 1983-2013 (n = 3454 chicks). Models with significant covariate effects are marked in bold characters. k = number of parameters, Dev = deviance, AIC = Akaike Information Criterion. cst = constant, dif = age difference between the parents, a.sex = interaction between age difference between the parents and sex of the juvenile. Best supported models are in bold characters.

| No. | Model | k | DEV | | AIC | | Slope **±** s.e. | |
| --- | --- | --- | --- | --- | --- | --- | --- | --- |
| *Survival* |  |  |  | |  | |  | |
| M1 | $\varphi(cst)$ | 68 | 20963.6 | | 21099.6 | |  | |
| **M2** | $\boldsymbol{\varphi(dif)}$ | **69** | **20958.6** | | **21096.6** | | **-0.11 ± 0.06** | |
| M3 | $\varphi(dif.sex)$ | 70 | 20958.5 | | 21098.5 | |  | |
| M4 | $\varphi(dif+\mathrm{dif}^{2})$ | 70 | 20958.6 | | 21098.6 | |  | |
| *Recruitment* |  |  | |  | |  | |  |
| **M9** | $\boldsymbol{\psi}^{\mathbf{rate}}\mathbf{(cst)}$ | **68** | **20963.6** | | **21099.6** | |  | |
| M10 | $\psi^{\mathrm{rate}}(dif)$ | 69 | 20961.8 | | 21099.7 | |  | |
| M11 | $\psi^{\mathrm{rate}}(dif.sex)$ | 70 | 20959.8 | | 21099.8 | |  | |
| M12 | $\psi^{\mathrm{rate}}(dif+\mathrm{dif}^{2})$ | 70 | 20958.6 | | 21098.6 | |  | |
| *Early recruitment* |  |  |  | |  | |  | |
| **M17** | $\boldsymbol{\psi}^{\mathbf{early}}\mathbf{(cst)}$ | **69** | **20962.2** | | **21100.2** | |  | |
| M18 | $\psi^{\mathrm{early}}(dif)$ | 70 | 20961.9 | | 21101.9 | |  | |
| M19 | $\psi^{\mathrm{early}}(dif.sex)$ | 71 | 20960.3 | | 21102.3 | |  | |
| M20 | $\psi^{\mathrm{early}}(dif+\mathrm{dif}^{2})$ | 71 | 20960.7 | | 21102.7 | |  | |

Online Table S5: Testing for the effects of the average age of both parents (a) on early life survival (φ), recruitment rate (ψ^rate^) and early recruitment probability (ψ^early^) for wandering albatrosses, Possession Island, 1983-2013 (n = 3454 chicks). k = number of parameters, Dev = deviance, AIC = Akaike Information Criterion. cst = constant, $\bar{a}$ = average parental age, $\bar{a}$.sex = interaction between average parental age and sex of the juvenile. Best supported models are in bold characters.

| No. | Model | | k | DEV | | AIC | |
| --- | --- | --- | --- | --- | --- | --- | --- |
| *Survival* |  | |  |  | |  | |
| **M1** | $\boldsymbol{\varphi(cst)}$ | | **68** | **20972.1** | | **21108.1** | |
| M2 | $\varphi(\bar{a})$ | | 69 | 20970.8 | | 21108.8 | |
| M3 | $\varphi(\bar{a}.sex)$ | | 70 | 20970.5 | | 21110.5 | |
| M4 | $\varphi(\bar{a}+\bar{a}^{2})$ | | 70 | 20970.1 | | 21110.1 | |
| *Recruitment* |  |  | | |  | |  |
| **M9** | $\boldsymbol{\psi}^{\mathbf{rate}}\mathbf{(cst)}$ | | **68** | **20972.1** | | **21108.1** | |
| M10 | $\psi^{\mathrm{rate}}(\bar{a})$ | | 69 | 20971.1 | | 21109.1 | |
| M11 | $\psi^{\mathrm{rate}}(\bar{a}.sex)$ | | 70 | 20970.8 | | 21110.8 | |
| M12 | $\psi^{\mathrm{rate}}(\bar{a}+\bar{a}^{2})$ | | 70 | 20970.1 | | 21110.1 | |
| *Early recruitment* |  | |  |  | |  | |
| **M17** | $\boldsymbol{\psi}^{\mathbf{early}}\mathbf{(cst)}$ | | **69** | **20971.0** | | **21109.0** | |
| M18 | $\psi^{\mathrm{early}}(\bar{a})$ | | 70 | 20970.9 | | 21110.9 | |
| M19 | $\psi^{\mathrm{early}}(\bar{a}.sex)$ | | 71 | 20970.8 | | 21112.8 | |
| M20 | $\psi^{\mathrm{early}}(\bar{a}+\bar{a}^{2})$ | | 71 | 20970.6 | | 21112.6 | |

Online Table S6: Testing for the simultaneous paternal ($a_{p}$) and maternal age effects on early life survival (φ) for wandering albatrosses, Possession Island, 1977-2013 (n = 5378 chicks). k = number of parameters, Dev = deviance, AIC = Akaike Information Criterion. cst = constant, $a_{p}$ = paternal age, $a_{m}$ maternal age, “+”= additive effect. The best supported model is in bold characters.

| No. | Model | k | DEV | AIC |
| --- | --- | --- | --- | --- |
| M1 | $\varphi(cst)$ | 68 | 35505.5 | 35641.5 |
| **M2** | $\boldsymbol{\varphi(}\mathbf{a}_{\boldsymbol{p}}\mathbf{)}$ | **69** | **35495.8** | **35633.8** |
| M3 | $\varphi(a_{p}+a_{m})$ | 70 | 35494.1 | 35634.1 |

Online Table S7: Testing for predominant paternal age effect on early life survival (φ) using interaction between parental age and the sex of the parent for wandering albatrosses, Possession Island, 1977-2013 (n = 5378 chicks). For chick from tow known-aged parents, we chose randomly paternal or maternal age. k = number of parameters, Dev = deviance, AIC = Akaike Information Criterion. cst = constant, $a$ = parental age, $\mathrm{sex}$ = sex of the parent, “.” = interaction effect. The best supported model is in bold characters.

| No. | Model | k | DEV | AIC |
| --- | --- | --- | --- | --- |
| M1 | $\varphi(cst)$ | 68 | 35505.5 | 35641.5 |
| **M2** | $\boldsymbol{\varphi(a)}$ | **69** | **35500.0** | **35638.0** |
| M3 | $\varphi(a.sex)$ | 70 | 35596.8 | 35636.8 |

Online Table S8: Investigating the relative importance of within-subject effects and between-subject effects on the relationships between parental age and early life survival (φ), recruitment rate (ψ^rate^) and early recruitment probability (ψ^early^) for wandering albatrosses, Possession Island, 1983-2013 (n = 5378 chicks). Following van de Pol and Wright (2009), we estimated the within-subject effects ($\beta_{W}$) and the between-subject effect ($\beta_{B})$. Within-subject effect is estimated though the relationships between the early-life demographic traits and a new parental age variable, $\Delta a$. To compute this variable for each chick, we subtracted the mean age of reproduction of the parent from the age of the parent at which the chick was raised, i.e. for a chick raised at occasion i by a parent j, $\Delta a_{ij}$ = $a_{ij}$ - $\bar{a}_{j}$. This procedure, called within-subject centering, eliminates any between-subject variation. Simultaneously, to estimate between-subject variation, we assessed the relationships between the early-life demographic traits and the average reproductive age of their parent ($\bar{a}_{j})$. k = number of parameters, Dev = deviance, AIC = Akaike Information Criterion. cst = constant, $\beta_{W}$ = the within-subject effects and $\beta_{B}$ = the between-subject effect. The best supported model is in bold characters.

| No. | Model | k | DEV | AIC | $\beta_{W}$ [95%CI] | $\beta_{B}$ [95%CI] |
| --- | --- | --- | --- | --- | --- | --- |
| *Survival* | |  |  |  |  |  |
|  | *Paternal age* |  |  |  |  |  |
| M1 | $\varphi(cst)$ | 68 | 28595.4 | 28731.4 |  |  |
| **M2** | $\boldsymbol{\varphi(\Delta a+}\bar{\mathbf{a}}\mathbf{)}$ | **70** | **28573.5** | **28713.5** | **-0.26 [-0.37;-0.15]** | **+0.01 [-0.08;+0.10]** |
|  | *Maternal age* |  |  |  |  |  |
| **M3** | $\boldsymbol{\varphi(cst)}$ | **68** | **27845.2** | **27981.2** |  |  |
| M4 | $\varphi(\Delta a+\bar{a})$ | 70 | 27840.6 | 27982.6 | -0.10 [-0.22;+0.02] | +0.03 [-0.07;+0.14] |
| *Recruitment* | |  |  |  |  |  |
|  | *Paternal age* |  |  |  |  |  |
| **M5** | $\boldsymbol{\psi}^{\mathbf{rate}}\mathbf{(cst)}$ | **68** | **28595.4** | **28731.4** |  |  |
| M6 | $\psi^{\mathrm{rate}}(\Delta a+\bar{a})$ | 70 | 28595.4 | 28737.4 | -0.05 [-0.16;+0.06] | +0.03 [-0.06;+0.13] |
|  | *Maternal age* |  |  |  |  |  |
| **M7** | $\boldsymbol{\psi}^{\mathbf{rate}}\mathbf{(cst)}$ | **68** | **27845.2** | **27981.2** |  |  |
| M8 | $\psi^{\mathrm{rate}}(\Delta a+\bar{a})$ | 70 | 27839.5 | 27981.5 | -0.03 [-0.14;+0.07] | +0.09 [-0.01;+0.19] |
| *Early recruitment* | | |  |  |  |  |
|  | *Paternal age* |  |  |  |  |  |
| **M9** | $\boldsymbol{\psi}^{\mathbf{early}}\mathbf{(cst)}$ | **69** | **28597.2** | **28735.2** |  |  |
| M10 | $\psi^{\mathrm{early}}(\Delta a+\bar{a})$ | 71 | 28594.5 | 28736.5 | -0.06 [-0.23;+0.10] | +0.03 [-0.12;+0.19] |
|  | *Maternal age* |  |  |  |  |  |
| **M11** | $\boldsymbol{\psi}^{\mathbf{early}}\mathbf{(cst)}$ | **69** | **27847.7** | **27985.7** |  |  |
| M12 | $\psi^{\mathrm{early}}(\Delta a+\bar{a})$ | 71 | 27846.2 | 27990.2 | -0.01 [-0.18;+0.17] | -0.03 [-0.20;+0.14] |
